# Commentary: Evaluation of Models of Parkinson's Disease

**DOI:** 10.3389/fnins.2016.00161

**Published:** 2016-04-19

**Authors:** Patricia Muñoz, Irmgard Paris, Juan Segura-Aguilar

**Affiliations:** ^1^Molecular and Clinical Pharmacology, Faculty of Medicine, University of ChileSantiago, Chile; ^2^Departamento de Ciencia Básicas, Facultad de Ciencias, Universidad Santo TomasViña del Mar, Chile

**Keywords:** preclinical models, Parkinson therapy, aminochrome, dopamine, neurodegeneration, MPTP, 6-hydroxidopamine, L-dopa

This publication is a review on the preclinical model used today for Parkinson's disease that take in consideration both preclinical model based on neurotoxin or mutations associated to familial Parkinson's disease (PD). The aim of this commentary is to introduce a new point of view about the actual preclinical models, open discussion, and propose a new preclinical model. The use of correct preclinical model to study the mechanisms and to test possible new therapies is essential to obtain successful results and new therapies. The discovery that dopamine loss was associated to the disease was essential to the use of L-dopa in Parkinson's disease therapy. L-dopa has been the most used drug in the disease treatment during near five decades despite the severe side effects observed after 4–6 years treatment (Segura-Aguilar et al., 2015). The actual treatment of the disease is based on dopaminergic and anti-cholinergics compounds. The scientific community and pharmaceutical companies have failed to find new drugs to halt the progression of the disease and the worst is that the focus to find new therapies is centered on drugs to alleviate the side effects of L-dopa such as dyskinesia. There are a long list of successful preclinical studies that have failed to translate these positive results to clinical studies and new therapies, for example pioglitazone, topiramate, GDNF, creatine, ubiquinone, cognane, mitoquinone, etc. (Athauda and Foltynie, 2015; Lindholm et al., 2015; Olanow et al., 2015; Park and Stacy, 2015).

The question is why successful results in preclinical studies cannot be translated to clinical studies? In our opinion (i) The preclinical models based on exogenous neurotoxins such as 6-hydroxidopamine and 1-methyl-4-phenyl-1,2,3,6-tetrahydropyridine (MPTP) has been very useful and valuable tools to study mechanisms. However, these preclinical models do not reflect what happen in the disease and therefore, these models are worthless to develop new drugs. One of the features of these exogenous neurotoxins is that all induce a rapid and extensive degeneration in animals. In humans MPTP induced a severe Parkinsonism in just 3 days after the use of a synthetic illicit drugs contaminated with MPTP. The rapid and extensive degeneration induced by exogenous drugs contrast with the very slow degeneration in Parkinson where the take years to develop motor symptoms. How we can translate results from preclinical to clinical studies when the mode of action is complete different. The exogenous drugs are killing all possible dopaminergic neurons which have affinity, suggesting that the very slow degeneration observed in Parkinson's disease must be dependent on an endogenous neurotoxin. It has been reported a new preclinical model based on β-sitosterol β-D-glucoside that triggers the progressive development of Parkinsonism, exhibiting olfactory deficit in the absence of significant cell loss or locomotor deficits. The question is whether this preclinical model can be used to develop new drugs in PD (Van Kampen et al., 2015); and (ii) the molecular mechanism(s) responsible for the loss of dopaminergic neurons containing neuromelanin remain unknown. The discovery of genes associated a familial form of Parkinson's disease do not explain what happen in the sporadic form of the disease but they resulted in an enormous input in basic research in order to understand the role of these proteins in the disease. In general there is an agreement in the scientific community that the neurodegeneration of dopaminergic neurons containing neuromelanin involves mitochondrial dysfunction, formation of neurotoxic alpha-synuclein oligomers, protein degradation dysfunction of both lysosomal and proteasomal systems, oxidative stress, neuroinflammation, and endoplasmic reticulum stress (Segura-Aguilar et al., 2014).

We have proposed that aminochrome can be used as a preclinical model for Parkinson disease (Segura-Aguilar et al., 2014, 2015). Unilateral injection of aminochrome into rat striatum induces a progressive contralateral behavior without loss of nigrostriatal dopaminergic neurons. Instead, aminochrome induces neuronal dysfunction of dopaminergic neurons since the level of dopamine significantly decrease while GABA level significantly increase, generating a neurotransmitter imbalance. The decrease in dopamine release without degeneration of nigrostriatal neurons can be explained by the fact that aminochrome induces mitochondrial dysfunction, significant decrease of ATP level in the striatum and in the number of synaptic vesicles (Herrera et al., 2016). Aminochrome is one of the *o*-quinones formed during dopamine oxidation to neuromelanin and it has been found to induce mitochondrial dysfunction (Aguirre et al., 2012), formation of neurotoxic alpha-synuclein oligomers (Muñoz et al., 2015), oxidative stress (Arriagada et al., 2004), dysfunction of protein degradation of both proteasomal (Zafar et al., 2006) and lisosomal (Huenchuguala et al., 2014) systems, endoplasmic reticulum stress (Xiong et al., 2014).

Aminochrome is the most stable *o*-quinone formed during dopamine oxidation and it participates on both neurotoxic and neuroprotective reactions. Aminochrome is neurotoxic when it forms adducts with proteins or it is one-electron reduced by flavoenzymes which catalyzes one electron transfer. However, there are two enzymes that prevent aminochrome neurotoxicity (Figure 1): (i) DT-diaphorase prevents aminochrome-induced cell death, mitochondrial dysfunction, oxidative stress, protein degradation dysfunction of both proteasomal and lysosomal systems, and formation of alpha-synuclein neurotoxic oligomers (Segura-Aguilar et al., 2014, 2015); and (ii) glutathione transferase M2-2 (GSTM2) catalyzes the GSH conjugation of both aminochrome and its precursor dopamine *o*-quinone and it is expressed only in astrocytes. GSTM2 prevent aminochrome toxicity in astrocytes when dopamine takes up from intersynaptic space after neurotransmission and oxidizes to aminochrome in the astrocytes. Interestingly, astrocytes secrete GSTM2 into conditioned medium and dopaminergic model neurons take up GSTM2 and prevent aminochrome-induced neurotoxicity in these neurons (Cuevas et al., 2015). Therefore, GSTM2 play a protective role against aminochrome both in astrocytes and dopaminergic neurons. The protective role of both DT-diaphorase and GSTM2 against aminochrome can explain why dopamine oxidation to neuromelanin is not normally a harmful pathway. The use of animals with DT-diaphorase or GSTM2 knockout injected with aminochrome maybe a new preclinical to study both PD mechanisms and to develop new PD drugs. In conclusion, the failure to translate successful result from preclinical to clinical studies and develop new pharmacological therapies will continue until we found a preclinical model that use an endogenous neurotoxin which is involved in the disease development.

**Figure 1:**
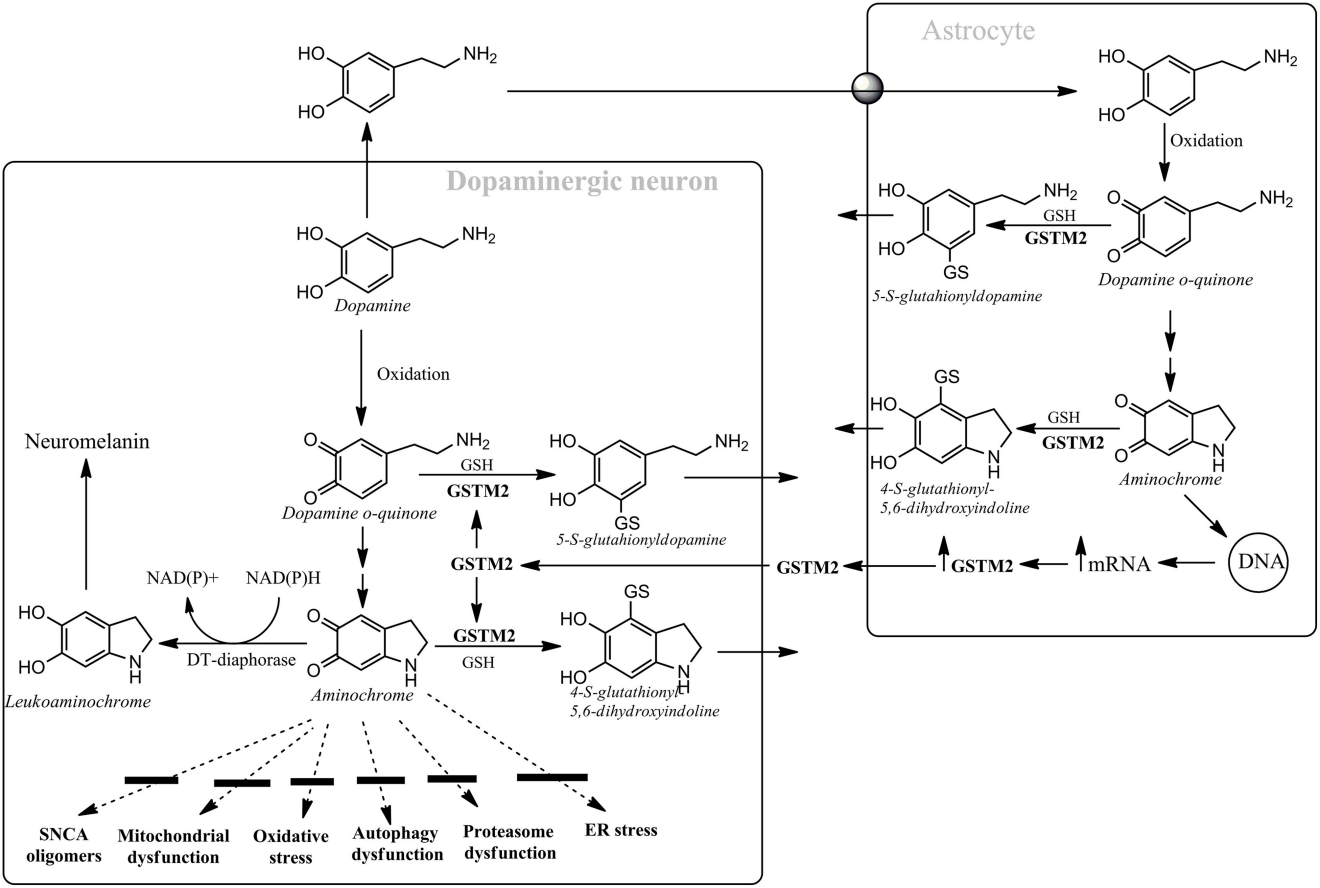
**Astrocytes protect dopaminergic neurons against aminochrome neurotoxicity**. Astrocytes secrete GSTM2 which is internalized by dopaminergic neurons in order to increase their protection against aminochrome. Dopamine oxidation to neuromelanin is a harmless pathway due to the presence of DT-diaphorase and GSTM2 that prevent aminochrome-dependent neurotoxicity by inhibiting the formation of alpha-synuclein (SNCA) neurotoxic oligomers, mitochondrial dysfunction, oxidative stress, autophagy, and proteasome dysfunction and endoplasmic reticulum stress.

## Author contributions

PM: search literature, critical review, final approval, agreement with all manuscript content. IP: interpretation of literature, critical review, final approval, and agreement with all manuscript content. JS: write the manuscript, critical review, final approval, and agreement with all manuscript concepts.

## Funding

Supported by University of Chile (ENL014/14).

### Conflict of interest statement

The authors declare that the research was conducted in the absence of any commercial or financial relationships that could be construed as a potential conflict of interest.
