# Corrigendum: Commentary: Evaluation of Models of Parkinson's Disease

**DOI:** 10.3389/fnins.2016.00320

**Published:** 2016-07-07

**Authors:** Patricia Muñoz, Irmgard Paris, Juan Segura-Aguilar

**Affiliations:** ^1^Molecular and Clinical Pharmacology, Faculty of Medicine, University of ChileSantiago, Chile; ^2^Departamento de Ciencia Básicas, Facultad de Ciencias, Universidad Santo TomasViña del Mar, Chile

**Keywords:** dopamine o-quinone, dopamine, aminochrome, neurodegenration, astrocytes, dopaminergic neurons

It was an error in the structure of dopamine o-quinone published in the commentary on Evaluation of models of Parkinson's disease since the benzene ring contained an extra double bound between the carbonyls. The actual structure of dopamine o-quinone is the correct structure.

**Figure 1:**
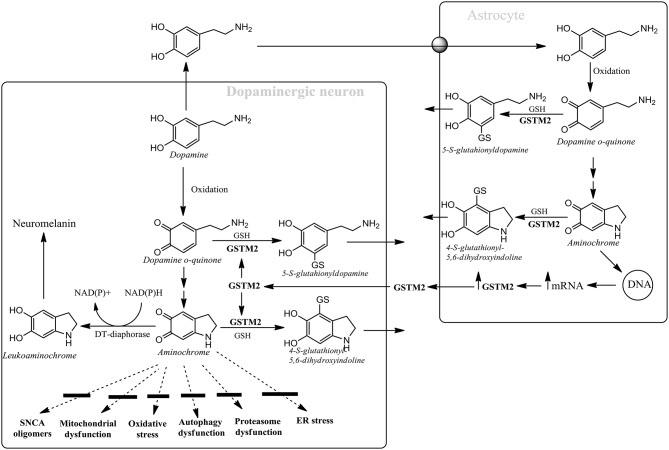
**Astrocytes protect dopaminergic neurons against aminochrome neurotoxicity**. Astrocytes secrete GSTM2 which is internalized by dopaminergic neurons in order to increase their protection against aminochrome. Dopamine oxidation to neuromelanin is a harmless pathway due to the presence of DT-diaphorase and GSTM2 that prevent aminochrome-dependent neurotoxicity by inhibiting the formation of alpha-synuclein (SNCA) neurotoxic oligomers, mitochondrial dysfunction, oxidative stress, autophagy, and proteasome dysfunction and endoplasmic reticulum stress.

## Author contributions

All authors listed, have made substantial, direct and intellectual contribution to the work, and approved it for publication.

## Funding

Supported by University of Chile (ENL014/14).

### Conflict of interest statement

The authors declare that the research was conducted in the absence of any commercial or financial relationships that could be construed as a potential conflict of interest.

